# Supplementary figures and images for: “We Should Be Working Together, and It Felt like They Disrupted That”: Pregnant Women and Partners’ Experiences of Maternity Care in the First UK COVID-19 Pandemic Lockdown
Source: Int J Environ Res Public Health. 2023 Feb 15;20(4):3382. doi: 10.3390/ijerph20043382 (PMC9962545; doi:10.3390/ijerph20043382)

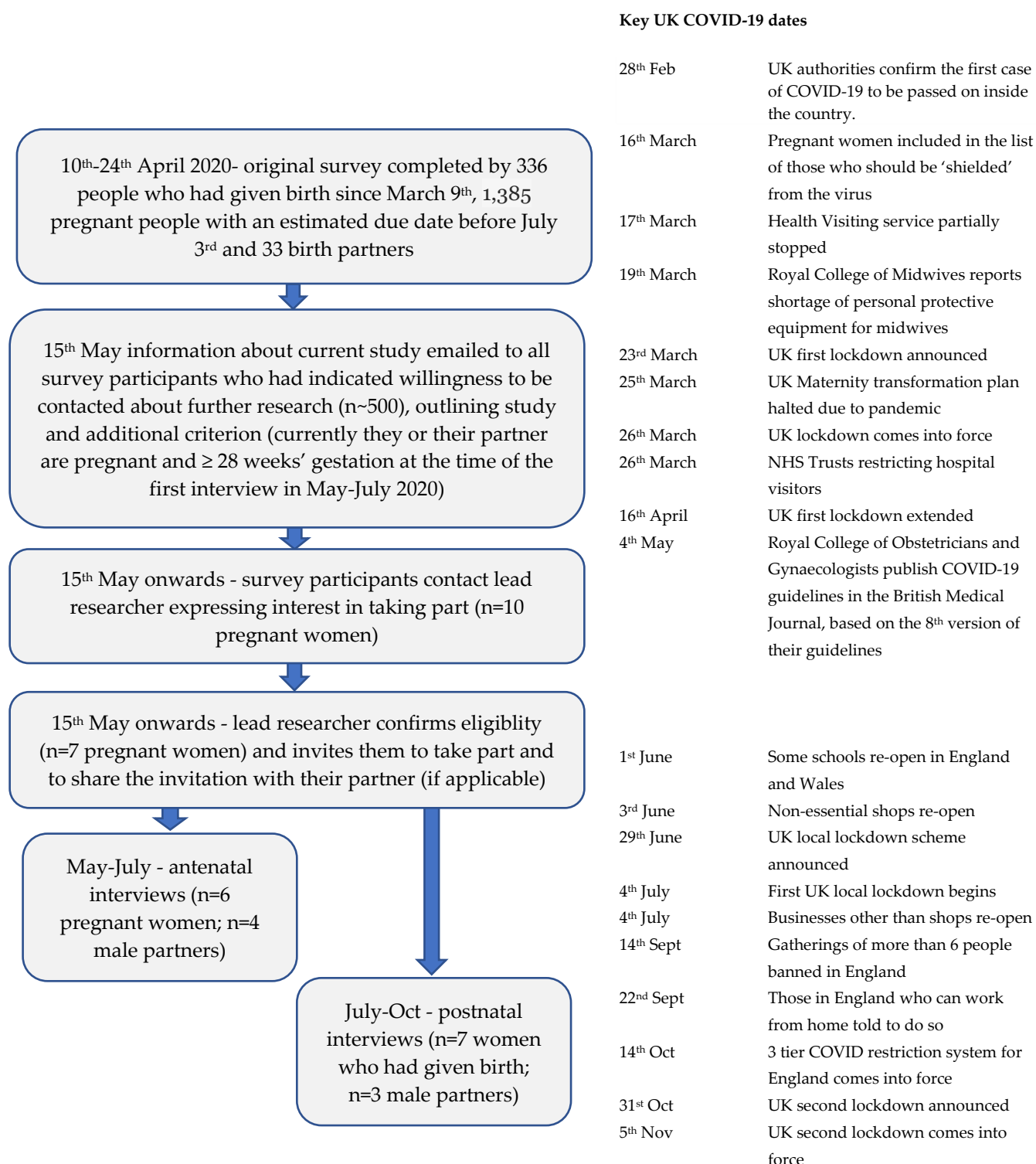

**Figure S1: Participant flow chart indicating timescales and associated restrictions in the UK**

Supplement: Supplementary file 1 [file ijerph-20-03382-s001.zip › ijerph-2181464-supplementary.pdf]
